# Metabolite annotations based on the integration of mass spectral information

**DOI:** 10.1111/j.1365-313X.2008.03434.x

**Published:** 2008-04-14

**Authors:** Yoko Iijima, Yukiko Nakamura, Yoshiyuki Ogata, Ken'ichi Tanaka, Nozomu Sakurai, Kunihiro Suda, Tatsuya Suzuki, Hideyuki Suzuki, Koei Okazaki, Masahiko Kitayama, Shigehiko Kanaya, Koh Aoki, Daisuke Shibata

**Affiliations:** 1 Kazusa DNA Research InstituteKazusa-Kamatari 2-6-7, Kisarazu 292-0818, Japan; 2 Ehime Women's CollegeIbuki, Uwajima 798-0025, Japan; 3 Graduate School of Information Science, Nara Institute of Science and TechnologyTakayama 8916-5, Ikoma, Nara 630-0101, Japan

**Keywords:** LC-FTICR-MS, metabolite annotations, secondary metabolites, *Solanum lycopersicum*, tomato

## Abstract

A large number of metabolites are found in each plant, most of which have not yet been identified. Development of a methodology is required to deal systematically with unknown metabolites, and to elucidate their biological roles in an integrated ‘omics’ framework. Here we report the development of a ‘metabolite annotation’ procedure. The metabolite annotation is a process by which structures and functions are inferred for metabolites. Tomato (*Solanum lycopersicum* cv. Micro-Tom) was used as a model for this study using LC-FTICR-MS. Collected mass spectral features, together with predicted molecular formulae and putative structures, were provided as metabolite annotations for 869 metabolites. Comparison with public databases suggests that 494 metabolites are novel. A grading system was introduced to describe the evidence supporting the annotations. Based on the comprehensive characterization of tomato fruit metabolites, we identified chemical building blocks that are frequently found in tomato fruit tissues, and predicted novel metabolic pathways for flavonoids and glycoalkaloids. These results demonstrate that metabolite annotation facilitates the systematic analysis of unknown metabolites and biological interpretation of their relationships, which provide a basis for integrating metabolite information into the system-level study of plant biology.

## Introduction

Large-scale biology studies supported by high-throughput data acquisition technologies require a method to bridge the gap between the data obtained and their biological interpretation. In genomics, without an analytical method to define genes, the nucleotide sequence of a whole genome is merely a series of letters (Ashburner, 2000). Using the process of annotation, by which information about the location and the number of genes and the functions of encoded proteins is inferred, researchers obtain biological meaning from the genome sequence (Stein, 2001). Metabolomics researchers are currently experiencing a similar situation to that which faced early genomics researchers. Recent progress in data acquisition technologies such as chromatography-coupled mass spectrometry has facilitated simultaneous detection and quantification of a large number of metabolite-derived peaks (Hall, 2006). However, the data obtained by high-throughput MS are merely a series of peaks without metabolite assignment. At this stage in metabolomics research, most of the peaks detected using MS cannot be assigned to identified metabolites. Such peaks are labeled as ‘unknown’ and usually are not characterized further. Thus the limited capability for metabolite identification has been one of the major obstacles in metabolomics (Kind and Fiehn, 2006; Wagner *et al.*, 2003).

One approach to overcoming this obstacle is to quantify all detected peaks and compile them as un-annotated variables (Bino *et al.*, 2005; Roessner *et al.*, 2001; Schauer *et al.*, 2005). This approach, non-targeted metabolic profiling, is frequently combined with statistical correlation analysis to hypothesize biological roles for the detected metabolites (Carrari *et al.*, 2006; Schauer *et al.*, 2006).

Another approach to overcoming the obstacle is to create a comprehensive dataset of plant metabolites by compiling various pieces of chemical information as has been done for human metabolites (Smith *et al.*, 2005), and to provide annotations for the metabolites. FTICR-MS is a promising candidate technology to achieve this goal. FTICR-MS measurement provides mass values with very high accuracy and resolution. This technology has been employed for non-targeted analyses of metabolites, and has demonstrated its advantage in detecting differentially expressed metabolites (Aharoni *et al.*, 2002; Murch *et al.*, 2004; Oikawa *et al.*, 2006). However, despite many technical advantages, FTICR-MS has a drawback in that it is incapable of separating isomers that have the same elemental compositions. It has been demonstrated recently that coupling of liquid chromatography to FTICR-MS facilitates the effective separation of isomers (Suzuki *et al.*, 2008). However, a comprehensive metabolite dataset using chromatography-coupled FTICR-MS has not yet been produced.

In the present study, we propose a procedure for metabolite annotation using the data obtained by high-performance LC-FTICR-MS. Tomato (*Solanum lycopersicum* cv. Micro-Tom) fruit was analyzed as a model plant for two reasons. First, tomato contains a number of secondary metabolites that are not present in other model plants such as Arabidopsis and rice. Second, a tomato genome sequencing project is currently underway (Mueller *et al.*, 2005) that will allow interpretation of metabolite data in conjunction with annotated gene functions.

Tomato metabolite data were collected in a non-targeted manner. We then compiled a dataset comprised of mass spectral features including retention time, UV/visible absorption spectrum, *m*/*z* value, *m*/*z* value of the MS/MS fragment, and relative intensity of the MS/MS fragment. These mass spectral features were attached as annotations to individual metabolites. This information allowed us to provide annotations of predicted molecular formulae for 869 metabolites. Comparison with public databases suggests that 494 of the metabolites are novel. Additionally, MS/MS fragmentation profile data allowed provision of annotations for a number of secondary metabolites with known chemical structures. We constructed a web-based database compiling the metabolite annotations (http://webs2.kazusa.or.jp/komics/). Based on comprehensive characterization of tomato fruit metabolites, we identified chemical building blocks that appear frequently in the tomato fruit tissues. We also assigned several unknown flavonoids and glycoalkaloids to novel metabolic pathways based on the annotations of putative structures. These results demonstrate that metabolite annotation allows us to systematically analyze unknown metabolites and facilitates biological interpretation of their roles in metabolic processes.

## Results

### Procedure of metabolite annotation

**Figure 1:**
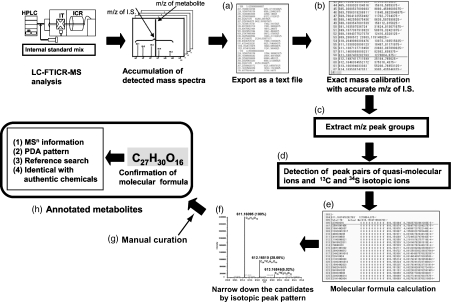
Schematic flow of the metabolite annotation procedure. (a) Raw data acquisition. (b) *m*/*z* calibration with internal standards. (c) Extraction of peak groups. (d) Isotopic ion assignment. (e) Molecular formula calculation. (f) Molecular formula screening using the relative intensity of isotopic ions. (g) Manual curation of isotopic, fragment and adduct peak assignment. (h) Provision of metabolite annotations. This procedure aims to identify a putative ‘metabolite’, which is defined as a group of mass signals that are detected in consecutive scans to form a peak group, accompanied by isotopic ions.

**Figure 2:**
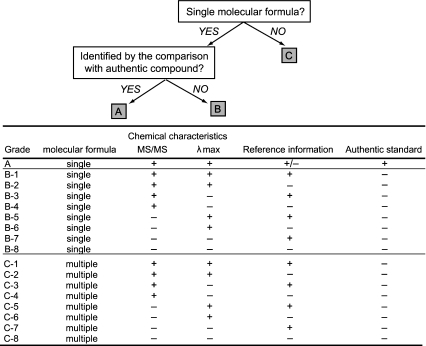
Annotation grading system. Metabolite annotations were classified according to the evidence that supports the annotations. Grade A consists of metabolites with annotations supported by comparison with authentic compounds. Grade B consists of metabolites with a single molecular formula. Grade C consists of metabolites with multiple molecular formulae. Grades B and C were divided into eight sub-grades according to the availability of MS/MS, λ_max_ and reference information.

We developed a procedure to organize MS data in a metabolite-oriented manner, which hereafter is referred to as a metabolite annotation procedure. The procedure comprises eight sequential steps. First, the whole raw data set comprising data from successive mass scans were exported as a text file (Figure 1a). Second, the observed *m*/*z* values of mass signals were calibrated with those of internal standards detected in the same scan (Oikawa *et al.*, 2006) (Figure 1b). After internal standard calibration, errors in *m*/*z* values decreased to less than 1 ppm (Table S1). Third, we grouped mass signals if the same *m*/*z* value was detected in consecutive scans, hereafter referred to as a ‘peak group’ (Figure 1c). An accurate *m*/*z* value for each peak group was calculated as the mean of the *m*/*z* values for the mass signals with the highest intensities (for details, see Experimental procedures). Fourth, we searched for pairs of peak groups that had *m*/*z* intervals (Δ) of 1.0033 and 1.9958 to identify ^12^C/^13^C_1_ isotopic peak pairs and ^32^S/^34^S_1_ isotopic peak pairs, respectively (Figure 1d). A peak group for the quasi-molecular ion accompanied by isotopic peaks was regarded as an individual ‘metabolite’. Fifth, molecular formulae were predicted from the accurate *m*/*z* values of the metabolites (Figure 1e). To avoid obtaining obviously unnatural formulae, we surveyed elemental compositions in the DNP database (Dictionary of Natural Products). Although the results for such a survey have been reported previously (Kind and Fiehn, 2007), we checked the maximum element numbers within our mass scan range (50–1500 Da). Our survey demonstrated that 95.65% of the DNP compounds (186 788 compounds in a range 50–1500 Da) consist of C, H, N, O, P and S within the ranges C 1–95, H 1–182, N 0–10, O 1–45, P 0–6 and S 0–5. Thus, we set these as upper limits for elemental compositions in the molecular formula calculations. Sixth, we narrowed down the number of candidate formulae using the relative intensity of the ^13^C_1_ and ^34^S_1_ isotopic ions (Figure 1f). A particular advantage of LC-FTICR-MS is that the resolution is high enough to separate the ^34^S_1_ isotopic ion from the ^13^C_2_ isotopic ion. Thus, we could use the relative intensity of the ^34^S_1_ isotopic ion as a constraint for the number of sulfur atoms. Seventh, we manually performed the isotopic peak group assignment and in-source fragment peak group assignment (Figure 1g). Assignment of the peak groups composed of adduct ions was also performed manually in this step. After these manual curation processes, metabolites were finally designated as ‘annotated metabolites’. In the eighth step, the mass spectral features (including retention time, *m*/*z* value, *m*/*z* value of the MS/MS fragment, relative intensity of the MS/MS fragment and UV/visible absorption spectrum) and database search results were attached to each metabolite as annotations (Figure 1h). All of the steps, except the manual curation process, are computerized. The annotated metabolites were classified using an annotation grading system (Figure 2, see Experimental procedures).

### Number of annotated metabolites in tomato fruit

We applied the metabolite annotation procedure to the MS data obtained from eight different tomato fruit tissues, comprising peel and flesh at the mature green, breaker, turning and the red stages. The number of detected mass signals ranged from 12 498 to 70 278 (Table 1). On average, 14.0 ± 3.6 mass signals were combined into one peak group. In both positive- and negative-ionization modes, 21 ± 1.7% of the peak groups were consistently assigned with the isotopic ions and recognized as metabolites. After manual curation, 57 ± 7.9% of the metabolites were provided with molecular formula annotations and designated as annotated metabolites. After removing the redundancy across samples, the total number of annotated metabolites was 869 (Table S2).

**Table 1:** The numbers of mass signals, peak groups, metabolites and annotated metabolites in tomato fruits

							Annotation grade		
							
Tissues	Ionization mode	Number of mass signals^a^	Number of peak groups^a^	Number of metabolites^a^	Number of annotated metabolites	Total number of annotated metabolites in each tissue^b^	A	B	C
Mature green									
Flesh	Positive	30 412 ± 3069	1470 ± 155	306 ± 35	154	267	13	146	108
	Negative	17 292 ± 1483	1673 ± 102	305 ± 22	167				
Peel	Positive	42 734 ± 5067	2311 ± 260	479 ± 69	228	368	18	184	166
	Negative	20 769 ± 2938	1925 ± 226	397 ± 51	228				
Breaker									
Flesh	Positive	28 782 ± 8835	1729 ± 271	357 ± 96	182	291	15	166	110
	Negative	15 853 ± 4078	1604 ± 311	308 ± 66	168				
Peel	Positive	43 462 ± 9540	2621 ± 379	636 ± 119	250	440	23	236	181
	Negative	32 675 ± 4440	2733 ± 376	602 ± 85	295				
Turning									
Flesh	Positive	24 353 ± 6111	1680 ± 58	352 ± 26	188	284	15	158	111
	Negative	12 498 ± 4924	1239 ± 460	251 ± 134	156				
Peel	Positive	63 258 ± 6645	3495 ± 348	784 ± 112	358	611	26	329	256
	Negative	39 274 ± 3449	3187 ± 364	676 ± 79	402				
Red									
Flesh	Positive	28 109 ± 1791	1700 ± 132	353 ± 42	179	263	18	147	98
	Negative	13 808 ± 4403	1444 ± 414	266 ± 64	144				
Peel	Positive	70 278 ± 3619	4305 ± 288	1039 ± 77	445	696	29	372	295
	Negative	55 429 ± 2452	4723 ± 301	1026 ± 68	428				

a Numbers indicate means ± SD of three measurements.

b Total numbers of non-redundant annotated metabolites detected in positive- and negative-ionization modes.

Only 3.6% of the metabolites were identified by comparison with authentic compounds (grade A, Table 1). Database searches in the DNP, KNApSAcK (Oikawa *et al.*, 2006), Kyoto Encyclopedia of Genes and Genomes (KEGG) (Goto *et al.*, 2002) and MotoDB (Moco *et al.*, 2006) revealed that 494 of the annotated metabolites were not present in the databases, suggesting that they are novel metabolites.

The complete set of LC-FTICR-MS data and metabolite annotations is accessible at http://webs2.kazusa.or.jp/komics/.

### Qualitative analysis of metabolite composition

**Figure 3:**
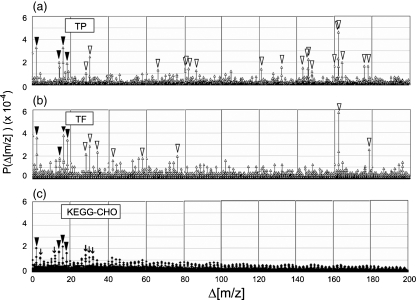
Examples of the distribution of Δ[*m*/*z* ] values in the 0–200 Da range at 0.001 Da intervals. Actual calculation of Δ[*m*/*z* ] values was performed in the 500 Da range. Δ[*m*/*z* ] values were calculated to obtain insights into the chemical building blocks that occur frequently in a set of metabolites. Δ[*m*/*z* ] values calculated from *m*/*z* values detected in positive-ionization mode from (a) peel at the turning stage (TP) and (b) flesh at the turning stage (TF), and (c) from the theoretical molecular weight of KEGG CHO compounds (KEGG-CHO). Closed arrowheads indicate Δ[*m*/*z* ] spikes that were detected in all three sample types (TP, TF and KEGG-CHO). Open arrowheads indicate Δ[*m*/*z* ] spikes that were observed specifically in tomato samples TP and TF. Arrows indicate Δ[*m*/*z* ] spikes that were observed specifically in KEGG-CHO. P(Δ[*m*/*z* ]) indicates the probability of the occurrence of Δ[*m*/*z* ] values.

Based on the metabolite annotations (Table S2), we investigated the distribution of mass differences between metabolites. Given that a metabolite is generated from a pre-existing metabolite by substitution of chemical building blocks, mass differences may provide insights into the types of reactions that have occurred between two metabolites. The distribution of Δ[*m*/*z* ] values showed ‘spikes’, demonstrating that certain Δ[*m*/*z* ] values occurred more frequently than others (Figure 3; the threshold probability to identify Δ[*m*/*z* ] spikes was determined as described in Figure S1). The Δ[*m*/*z* ] spike profiles seen in tomato fruit samples were different from those of 10 743 compounds containing C, H and O listed in KEGG (Goto *et al.*, 2002) (Figure 3c; for a complete list of the compounds, see Table S3). This demonstrates that the Δ[*m*/*z* ] spikes have a sample-specific profile. The Δ[*m*/*z* ] spikes that occurred in the tomato samples are listed in Table S4.

**Figure 4:**
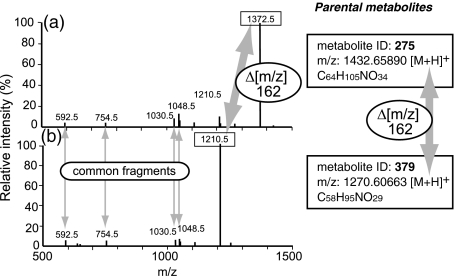
An example of the MS/MS spectra comparison to confirm biological relevance of Δ[*m*/*z* ] values. MS/MS spectra of metabolite ID 275 (a) and metabolite ID 379 (b). The MS/MS spectral data for metabolite ID 275 and metabolite ID 379 are provided at http://webs2.kazusa.or.jp/komics/. Comparison of (a) and (b) demonstrates that an Δ[*m*/*z* ] value between the two metabolites was observed in a pair of MS/MS fragments (*m/z* 1372.5 and *m/z* 1210.5), and that there are several MS/MS fragments with identical *m/z* values suggesting that Δ[*m/z* ] observed between metabolite ID 275 and ID 379 is biologically relevant.

We then checked whether Δ[*m*/*z* ] spikes were generated from biologically relevant metabolite pairs, i.e. that Δ[*m*/*z* ] values were produced in combinations that reflect reaction relationships. This was achieved by inspecting the MS/MS fragmentation data (available at http://webs2.kazusa.or.jp/komics/). Biologically relevant metabolite pairs were screened according to two criteria. First, the Δ[*m*/*z* ] value observed between the metabolites must be observed in more than one pair of MS/MS fragments. Second, metabolite pairs must have more than one identical MS/MS fragment. The relative intensity of the MS/MS fragment ions was not taken into account. For example, Figure 4 shows the MS/MS spectra of a pair of metabolites with *m*/*z* values of 1372.5 (Figure 4a) and 1210.5 (Figure 4b), with a Δ[*m*/*z* ] value of 162.053 between the fragments. In addition, several common fragments were detected in the MS/MS spectra of these two metabolites. Thus, the pair is regarded as biologically relevant. We manually inspected the MS/MS spectra of all 2722 metabolite pairs that contributed to the formation of Δ[*m*/*z* ] spikes, and found that approximately 37% was biologically relevant (Table S4). Further screening for biologically relevant metabolite pairs was performed by inspecting annotations of putative structures and database hits to determine whether occurrence of a Δ[*m*/*z* ] value was possible based on knowledge of the biochemical reactions. The Δ[*m*/*z* ] values with the highest percentages of relevant metabolite pairs include those corresponding to chemical building blocks C_3_H_7_NO_2_S (121.020), caffeic acid (162.032), hexose (162.053 and 162.054), malonic acid (86.001) and the amino group (17.027) (Table 2). The Δ[*m*/*z* ] spike profiles show tissue- and ripening stage-dependent differences (Figure S2). To confirm the ripening stage-dependent changes, Δ[*m*/*z* ] values between metabolites in two consecutive stages were analyzed (for details, see Experimental procedures). The analysis indicated that addition of chemical building blocks such as an amino group, caffeic acid, a C_3_H_7_NO_2_S moiety or hexose occurred frequently during ripening. According to the annotations of putative structure and database hits, these chemical building blocks are frequently associated with secondary metabolism.

**Table 2:** Biologically relevant Δ[*m*/*z* ] spikes estimated by inspection of MS/MS spectra, putative structures and database hits

	MS/MS inspection results			Elemental composition difference^c^	Putative chemical building blocks
			
Δ[*m*/*z* ] value	Relevant (%)	Not relevant (%)	No MS/MS (%)	Description	Description
121.020	97.3	0.0	2.7	C_3_H_7_NO_2_S	Addition of C_3_H_7_NO_2_S
456.149^a^	93.8	0.0	6.2	C_17_H_28_O_14_	NS^d^
162.032	63.9	0.0	36.1	C_9_H_6_O_3_	Addition of caffeic acid Hydroxylation and addition of coumaric acid
104.048	26.7	0.0	73.3	C_4_H_8_O_3_	NS^d^
143.277	76.5	2.9	20.6	Addition of C_12_H_33_N, and deletion of O_3_	NS^d^
162.053^b^	57.9	5.8	36.3	C_6_H_10_O_5_	Addition of hexose Hydroxylation and addition of deoxyhexose
86.001	67.9	10.3	21.8	C_3_H_2_O_3_	Addition of malonic acid
162.054^b^	60.0	9.1	30.9	C_6_H_10_O_5_	Addition of hexose Hydroxylation and addition of deoxyhexose
456.148^a^	50.0	9.1	40.9	C_17_H_28_O_14_	NS^d^
440.153	47.1	11.8	41.2	C_17_H_28_O_13_	NS^d^
17.027	45.2	14.3	40.5	H_3_N	Addition of an amino group
42.011	33.3	14.8	51.9	C_2_H_2_O	NS^d^

a Assigned to the same elemental composition, respectively.

b Assigned to the same elemental composition, respectively.

c Elemental composition difference with the highest percentage in all molecular formula combinations.

d Not suggested. Known chemical blocks were not suggested by putative structures or database hits.

### Secondary metabolites in tomato

In addition to the frequently occurring mass differences, the tomato fruit metabolites analyzed using LC-FTICR-MS include diverse flavonoids and glycoalkaloids. Of the 869 annotated metabolites, 70 and 93 were assigned to the flavonoid and glycoalkaloid groups, respectively. The number of flavonoids increased during ripening (Table S5). In addition, peel tissues contained a larger number of flavonoids than flesh. Four chalcone and flavanone aglycones [naringenin chalcone (NGC), naringenin (NG), eriodictyol (ED) and eriodictyol chalcone (EDC)] and two flavonol aglycones [kaempferol (Kae) and quercetin (Que)] were identified by MS/MS and MS^3^ fragmentation patterns combined with UV/visible absorption spectra, as reported previously (Bino *et al.*, 2005; Iijima *et al.*, 2008). Dehydrokaempferol glycosides, previously identified in other cultivars of tomato (Le Gall *et al.*, 2003; Moco *et al.*, 2006), were not detected in the Micro-Tom samples.

MS/MS fragmentation patterns of the flavonoids demonstrated the occurrence of various glycosylations and acylations. Flavonoids in the chalcone/flavanone and flavonol groups showed different conjugation patterns. Conjugate moieties of NH_3_ (*m*/*z* 17.027) and C_3_H_7_NO_2_S (*m*/*z* 121.020) were associated exclusively with chalcones and flavanones. On the other hand, deoxyhexose, *p*- coumaroyl hexose and feruloyl hexose were associated exclusively with Kae and Que.

**Figure 5:**
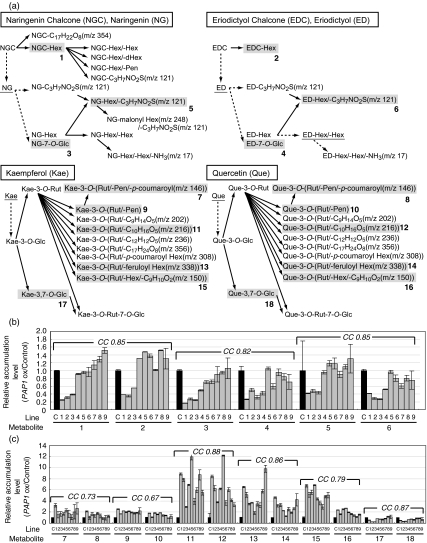
Reaction and pathway relationships of Micro-Tom flavanoids. (a) Putative metabolic pathway for the flavonoids. Underlined letters indicate metabolites that were not detected in this study. Solid arrows indicate the occurrence of modification between the detected metabolites. Broken arrows indicate possible reactions between detected and non-detected metabolites. Hex, hexose; dHex, deoxyhexose; Glc, glucose; Rut, rutinose; Pen, pentose. (b, c) Correlations between the relative accumulation levels of (b) chalcone/flavanone metabolites and (c) flavonol metabolites in Arabidopsis *PAP1*- over-expressing tomato fruits (gray bars) in comparison with control fruit (black bars). Lines: C, control; 1–9, independent lines of *PAP1*- over-expressing Micro-Tom. Metabolites: numbers indicate the metabolites shown in (a) (highlighted by gray shading). CC, correlation coefficient. Means ± SD of three biological repeats are indicated.

Possible pathway relationships for the flavonoids are illustrated based on the putative structures (Figure 5a). The modification pattern observed in the NGC pathway is quite similar to that in the EDC pathway. Likewise, the modification patterns observed in pathways starting from Kae and Que are similar to each other. The apparent similarities suggest that regulation of modification reactions may be similar between the NGC and EDC pathways and between the Kae and Que pathways. To test this, we investigated flavonoid levels in fruits of transgenic Micro-Tom lines over-expressing *PAP1*, an Arabidopsis transcription factor that up-regulates flavonoid pathway genes (Borevitz *et al.*, 2000). We focused on comparison of the pairs of NGC and EDC derivatives and the pairs of Kae and Que derivatives, each of which has an identical conjugate moiety (numbered metabolites in Figure 5a). The accumulation levels of three pairs of metabolites in the NGC and EDC pathways changed in a highly correlated manner (correlation coefficient >0.6) in *PAP1* over-expressing lines (Figure 5b), as did those of six pairs of metabolites in the Kae and Que pathways (Figure 5c). This suggests that pairs of genes responsible for the same modification reactions are coordinately regulated by the over-expression of *PAP1*. Alternatively, each pair of modifications may be catalyzed by an identical enzyme.

**Figure 6:**
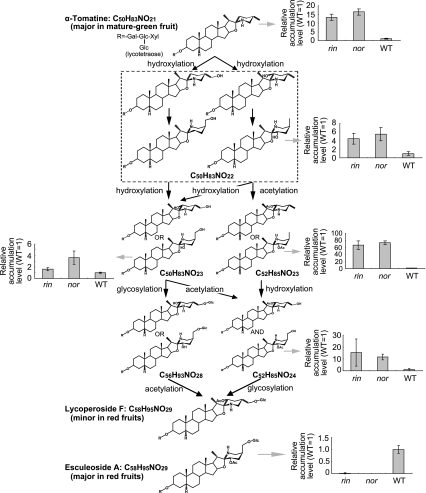
Putative metabolic pathway from α-tomatine to esculeoside A. Graphs show the relative abundance of indicated metabolites (gray arrows) in *nor* and *rin* mutant fruits (containing both peel and flesh) in comparison with wild-type Rutgers (WT), the background line of the mutants. Means ± SD of three biological repeats are indicated. Esculeoside A was almost absent in the fruits of *nor* and *rin* mutants. However, other intermediate glycoalkaloids accumulated at higher levels in *nor* and *rin* than WT. The result suggests that the final step of esculeoside A biosynthesis (glycosylation of C_52_H_85_NO_24_) is controlled by developmentally regulated ethylene production.

Most of the glycoalkaloids annotated in this study (Table S6) appear to be novel, as they were not found in the literature or public databases. The composition of glycoalkaloids showed tissue-dependent differences. Peel contained a larger number of glycoalkaloids than flesh. The composition of glycoalkaloids also appeared to change with ripening. The intensity of the mass peak of tomatine (*m*/*z* 1034.55303 [M+H]^+^) was high in fruits at the mature green and breaker stages, but very weak at the red stage, suggesting that levels of tomatine decreased during ripening. On the other hand, a number of glycoalkaloids that are larger than tomatine were detected at the red stage. According to MS^*n*^ data, some of these were assigned as putative intermediate metabolites in the metabolic pathway between tomatine and esculeoside A, the major glycoalkaloid at the red stage (Fujiwara *et al.*, 2004) (Figure 6). To test whether this pathway is regulated by ripening, we investigated the accumulation levels of the intermediates in fruit tissues (containing both peel and flesh) of *non-ripening* (*nor*) and *ripening-inhibitor* (*rin*) mutants that do not exhibit ripening-associated ethylene production. The levels of metabolites upstream of C_52_H_85_NO_24_ increased in *nor* and *rin* fruits in comparison with wild-type Rutgers, but the level of esculeoside A decreased remarkably (Figure 6). This indicates that the final step of esculeoside A biosynthesis is associated with developmentally regulated ripening events.

## Discussion

### Concept of metabolite annotation

We established a metabolite annotation procedure and constructed a comprehensive metabolite annotation database to organize experimental information obtained by LC-FTICR-MS, using tomato as a model plant species. The term ‘metabolite annotation’ has been proposed previously to describe the process of labeling experiments with biological metadata (such as a description of actual experimental conditions) in order to help unravel the biological role of metabolites based on changes in their levels in response to genetic and environmental perturbation (Fiehn *et al.*, 2005; Scholz and Fiehn, 2007). Their concept of ‘metabolite annotation’ comprises (i) mass spectral annotation and (ii) biological metadata annotation. In this study, we used the term ‘metabolite annotation’ to describe a procedure by which mass spectral information is provided to individual metabolites, thus our annotation procedure can be classified as mass spectral annotation.

The metabolite annotation procedure reported in this study is based on four novel concepts. First, we provided annotations to individual ‘metabolites’. We identified metabolite-representing peaks systematically based on the following criteria: (i) that mass signals were detected in consecutive scans to form a peak group, and (ii) that quasi-molecular ions were accompanied by isotopic ions. Second, we aimed to establish a data-driven annotation protocol for LC-MS-derived data as only a few metabolic profiling methods for LC-MS-derived data have been reported (De Vos *et al.*, 2007; Smith *et al.*, 2006). This is in contrast to the well-established metabolic profiling methods for GC-MS-derived data (Duran *et al.*, 2003; Fiehn *et al.*, 2005; Tikunov *et al.*, 2005). Third, we provided annotations for non-volatile secondary metabolites that are difficult to detect by GC-MS, which allowed us to explore a diverse range of secondary metabolites. Fourth, we introduced a grading system to describe the experimental evidence by which the annotation was supported. It should be mentioned that the metabolite annotations provided in this study are open to future curation. For example, heuristic rules for filtering molecular formulae have been proposed recently (Kind and Fiehn, 2007). In the current study, we implemented procedures equivalent to element number filtering, LEWIS and SENIOR checks, and isotopic pattern filtering, but did not implement element ratio checks or element probability checks. Thus, curation of molecular formula annotations will be feasible by applying these rules.

### Limitation in complete coverage and quantification of metabolites

In this study, tomato fruit tissues were extracted using 75% w/v methanol. This method was suitable for extracting a wide range of secondary metabolites, amino acids, sugars, nucleotides and organic acids, but did not extract non-polar metabolites such as lycopene. This demonstrates that the metabolite composition detected is inevitably biased by the choice of extraction method. Thus, an appropriate combination of multiple extraction methods is needed for complete coverage of metabolites.

For comprehensive profiling of the annotated metabolites, quantification depends on the measurement of mass signal intensity. However, differences in the mass signal intensity may be caused by a different degree of ion suppression, a phenomenon by which the intensity of a certain ion is suppressed by the presence of other ions. Even with LC separation prior to MS, several peaks co-eluted in single *m*/*z* scans. We performed semi-quantitative analyses of flavonoids and glycoalkaloids based on comparison of the relative mass signal intensities of an identical metabolite across samples (Figures 5b,c and 6). To minimize the possibility that mass signal intensity was affected by different degrees of ion suppression, we checked (i) whether the mass signal intensity is proportional to the UV/visible absorbance, (ii) whether the profile of ions co-eluted with the target ion is similar, and (iii) whether ion suppression is observed in the intensity of co-injected internal calibration standards. Further study is needed to estimate the extent to which ion suppression affects the quantification.

### Novel metabolites in tomato fruit

Comparison of 869 annotated metabolites with compounds registered in public databases revealed that 494 of the annotated metabolites appear to be novel. Putative structures for the novel metabolites can be predicted from the annotations of MS/MS fragmentation data. This was particularly effective in predicting putative structures of novel flavonoids and glycoalkaloids. In the flavonoid group, an unknown moiety, C_3_H_7_NO_2_S (*m*/*z* 121.020), was found as conjugates with NGC, NG and ED. Its predicted molecular formula matched that of cysteine. It has been reported that cysteine forms a conjugate with epicatechin when procyanidins depolymerize in the presence of cysteine (Torres *et al.*, 2002). However, cysteine conjugates of chalcones and flavanones have not been reported. Structural identification of the moiety will be required to understand the biosynthesis of C_3_H_7_NO_2_S conjugates. Modification of flavonoids has been attracting attention as the biological effects of flavonoid conjugates depend on the nature of the conjugate moieties. The tomato flavonoids found in the present study provide an experimental basis to search for novel functional flavonoids, and to elucidate unknown mechanisms of flavonoid modification. In the glycoalkaloid group, our results indicated the presence of novel glycoakaloids with *m*/*z* values larger than the maximum molecular mass (1271 Da) of tomato glycoalkaloid reported so far (Ono *et al.*, 2006) (Table S6). Most of these novel glycoalkaloids appeared after the onset of ripening. This suggests that glycoalkaloid metabolism is active during fruit ripening, and that glycoalkaloids play unidentified physiological roles in the ripening fruit.

Carotenoids, another major secondary metabolite group in tomato, were not detected under our experimental conditions. Development of a metabolite annotation method for MS data obtained in atmospheric pressure photo-ionization mode, which efficiently ionizes non-polar metabolites including carotenoids, is currently underway.

### Reaction and pathway relationships

Metabolite annotations aid our understanding of mechanisms controlling metabolism from chemical and biological points of view. From a chemical point of view, metabolite annotations provide detailed chemical information for each metabolite, which will serve as a basis for identifying unknown metabolites. From a biological point of view, metabolite annotations provide a basis for elucidating biological relationships between metabolites, such as reaction and pathway relationships.

To obtain insights into reaction relationships between metabolites, we performed mass difference analysis. Several Δ[*m*/*z* ] values occur frequently in metabolites from tomato fruit, suggesting that chemical building blocks corresponding to those Δ[*m*/*z* ] values appear frequently in tomato fruit metabolites. It should be emphasized that signal intensities were not taken into consideration in this analysis. Thus, when we state that certain Δ[*m*/*z* ] values occur frequently, this does not mean that the accumulation levels of these metabolites are high. Nevertheless, mass difference analysis combined with inspection of MS/MS spectra annotations provides an efficient way to study metabolites relating to a reaction of interest.

To understand the metabolic pathway relationships between annotated metabolites, we arranged flavonoids detected in this study into metabolic diagrams (Figure 5a). These demonstrate that the modification patterns between the NGC and EDC pathways and between the Kae and Que pathways, respectively, are similar to each other. When the flavonoid pathway was up-regulated by over-expression of *PAP1*, changes in the relative accumulation levels of several pairs of metabolites with identical conjugation patterns were highly correlated (Figure 5b,c). This result demonstrates that genes responsible for each pair of modification reactions are coordinately regulated by *PAP1*. Alternatively, identical enzymes may use both Kae and Que derivatives as substrates, as reported previously for flavonol glycosyltransferases (Jones *et al.*, 2003; Yonekura-Sakakibara *et al.*, 2007). For glycoalkaloids, a biosynthetic pathway from tomatine to esculeoside A (Fujiwara *et al.*, 2004) was illustrated (Figure 6). By analyzing fruits of *nor* and *rin* mutants, we have demonstrated that the reaction step between C_52_H_85_NO_24_ and esculeoside A is regulated by the occurrence of ripening, which is developmentally controlled by *NOR* and *LeMADS-RIN* (Giovannoni, 2004). These results demonstrate that the metabolite annotation procedure is a powerful approach for producing hypotheses with respect to unknown metabolic pathways.

### Possible link between metabolite annotations and integrated ‘omics’ study

Further insights into the regulation of metabolite biosynthesis will be obtained by the integration of metabolomics data with other ‘omics’ data. A parallel analysis of metabolites and transcripts is a promising approach to achieve this goal (Hirai *et al.*, 2004; Nikiforova *et al.*, 2005; Tohge *et al.*, 2005; Urbanczyk-Wochniak *et al.*, 2003). Another promising approach involves combination of metabolite analysis with genetic analysis such as quantitative trait loci (QTL) analysis (Keurentjes *et al.*, 2006; Morreel *et al.*, 2006; Schauer *et al.*, 2006). In such approaches, the metabolite annotation plays a complementary role to the metabolic profiling in linking metabolite information to other ‘omics’ information. By contrast to quantitative metabolic profiling, annotations of mass spectral features facilitate qualitative characterization with respect to identity, structural similarity and biochemical relationships between the metabolites. This assists in inference of biological meanings from metabolic profiling combined with other ‘omics’ data. Additionally, new metabolites predicted by the metabolite annotations will be included in multi-‘omics’ pathway tools (Thimm *et al.*, 2004; Tokimatsu *et al.*, 2005; Zhang *et al.*, 2005), and expand our knowledge about unknown metabolic pathways. Metabolite annotations provide firm foundations for integrating chemical information regarding metabolites into a system-level study of plant metabolism.

## Experimental procedures

### Plant materials

Seeds of cultivated tomato (*S. lycopersicum* cv. Micro-Tom) were sown in pots (500 ml) filled with a mixture of vermiculite and Powersoil (mix ratio 1:1, Kureha Chemical Industries, http://www.kureha.co.jp/ and Kanto Hiryou Industries, http://www.okumurashoji.co.jp/). Until germination, seeds were covered with plastic film and kept in the dark at 25°C. After 4 days in the dark, they were grown with a photoperiod of 16 h light (80 μmol m^−2^ s^−1^)/8 h dark at 25°C. Hyponex® (Hyponex Ltd, http://www.scotts.com/) at 1000-fold dilution was applied to plants once a week. Fruits at the mature green (G, approximately 30 days after anthesis), breaker (B, approximately 35 days after anthesis), turning (T, approximately 38–40 days after anthesis) and red (R, approximately 45–48 days after anthesis) stages were harvested. A vector construct expressing Arabidopsis *PAP1* under the control of the CaMV 35S promoter (Tohge *et al.*, 2005) was provided by K. Saito (Chiba University, Japan). Transformation of Micro-Tom was performed according to the protocol reported previously (Sun *et al.*, 2006). Seeds of wild-type Rutgers (LA1090) and the *nor* (LA3013) and *rin* (LA3012) mutants were obtained from the C.M. Rick Tomato Genetic Resource Center (University of California, Davis, CA, USA).

### Metabolite extraction

The peel and the flesh of tomato fruit were separated using a razor blade. Each sample was sliced, immediately frozen in liquid nitrogen and ground to powder using a Shake Master homogenizer (Biomedical Science, http://www.bmsci.com). Powdered samples (50–70 mg) were extracted with three volumes of methanol containing formononetin (20 μg ml^−1^) as an internal standard. After homogenization using a Mixer Mill MM 300 (Qiagen, http://www.qiagen.com/) at 27 Hz for 2 min twice, homogenates were centrifuged (12 000 ***g***, 10 min, 4°C). The supernatant was filtered through 0.2 μm PVDF membrane (Whatman, http://www.whatman.com), and the filtrate was used for LC-FTICR-MS analysis.

### LC-FTICR-MS analysis

An Agilent 1100 system (Agilent, http://www.agilent.com) coupled to a Finnigan LTQ-FT (Thermo Fisher Scientific; http://www.thermofisher.com) was used for LC-FTICR-MS analysis. The data were acquired and browsed using Xcalibur software version 2.0 (Thermo Fisher Scientific). Methanol extract was applied to a TSKgel column ODS-100V (4.6 × 250 mm, 5 μm; TOSOH Corporation, http://www.tosoh.com). Water (HPLC grade; solvent A) and acetonitrile (HPLC grade; solvent B) were used as the mobile phase with 0.1% v/v formic acid added to both solvents. The gradient program was as follows: 10% B to 50% B (50 min), 50% B to 90% B (20 min), 90% B (5 min) and 10% B (10 min). The flow rate was set to 0.5 ml min^−1^, and the column oven temperature was set at 40°C; 20 μl of each sample were injected.

To monitor HPLC elution, a photodiode array detector was used in the wavelength range 200–650 nm. The ESI setting was as follows: spray voltage 4.0 kV and capillary temperature 300°C for both positive- and negative-ionization modes. Nitrogen sheath gas and auxiliary gas were set at 40 and 15 arbitrary units, respectively. A full MS scan with internal standards was performed in the *m*/*z* range 100–1500 at a resolution of 100 000 (at *m*/*z* 400).

A mixture of internal calibration standards dissolved in 50% v/v acetonitrile was introduced by a post-column method at a flow rate of 20 μl min^−1^. The concentration of each standard in the mixture was as follows: for positive mode,: 10 μm lidocaine (*m*/*z* 235.18049 [M+H]^+^; Sigma-Aldrich, http://www.sigmaaldrich.com/), 5 μm prochloraz (*m*/*z* 376.03809 [M+H]^+^; AccuStandard Inc., http://www.accustandard.com), 1.2 μm reserpine (*m*/*z* 609.28066 [M+2H]^2+^; Sigma-Aldrich), 0.8 μm bombesin (*m*/*z* 810.41481 [M+H]^+^; Sigma-Aldrich), 0.4 μm aureobasidin A (*m*/*z* 1123.67778 [M+Na]^+^; Takara Bio Inc., http://www.takara-bio.com), 22 μm vancomycin (*m*/*z* 1448.43747 [M+H]^+^; MP Biomedicals Inc., http://www.mpbio.com); for negative mode: 11.2 μm 2,4-dichlorophenoxyacetic acid (*m*/*z* 218.96212 [M-H]^−^; Sigma-Aldrich), 3.1 μm ampicillin (*m*/*z* 348.10235 [M-H]^−^, Sigma-Aldrich), 0.25 μm CHAPS (*m*/*z*: 659.39468 [M+HCOO]^−^; Sigma-Aldrich), 1.0 μm tetra-*N*- acetylchitotetraose (*m*/*z* 875.32626 [M+HCOO]^−^; Toronto Research Chemicals, Inc., http://www.trc-canada.com), 0.6 μm aureobasidin A (*m*/*z* 1145.68676 [M+HCOO]^−^, Takara Bio Inc.). MS/MS and MS^3^ fragmentation were carried out at a normalized collision energy of 35.0% and a isolation width of 4.0 (*m*/*z*), and were obtained by ion trap mode. Relative accumulation levels of flavonoids and glycoalkaloids were estimated by dividing the peak area of the metabolite by that of internal standard (formononetin).

### Chemicals

Authentic naringenin chalcone was generously provided by the Kikkoman Corporation (http://www.kikkoman.com). Esculeosides A and B were kindly provided by T. Nohara and Y. Fujiwara (Kumamoto University, Japan). Other authentic compounds were purchased from EXTRASYNTHESE (http://www.extrasynthese.com), Funakoshi Co. Ltd (http://www.funakoshi.co.jp), Sigma-Aldrich, Tokyo Chemical Industry (http://www.tci-asiapacific.com) and Wako Pure Chemical Industries Ltd (http://www.wako-chem.co.jp/).

### Metabolite annotation procedure

A program written in Microsoft VC^++^ was used to export the raw data (XRAW) file of each single run as a text file. The output file includes retention time, scan number, *m*/*z* value and their intensities. To discriminate mass signals from baseline noise, mass signals whose intensities were more than three times the baseline level of each scan were selected. Next, *m*/*z* values of all ions in each scan were bulk-calibrated with observed *m*/*z* values of internal calibration compounds in the same scan using the computational tool DrDMASS (http://kanaya.naist.ac.jp/DrDMASS/, Oikawa *et al.*, 2006). By using internally calibrated *m*/*z*, if the*m*/*z* were obtained in more than 30% of the total mass scans, those mass signals could be regarded as artificial noise and thus excluded from further analyses. After removing noise, all data were collected as a Microsoft Excel file. The quasi-molecular ions detected with a ^13^C isotopic ion in the scan at an *m*/*z* value that was 1.003 greater were selected. After sorting mass signals by scan number, those detected in more than three consecutive scans were selected and grouped. If a peak group consisted of three or four mass signals, an accurate *m*/*z* value for the group was obtained as the mean *m*/*z* value for the three or four mass signals. If a peak group consisted of five or more mass signals, an accurate *m*/*z* value was obtained as the mean *m*/*z* value for the five most intense signals. For the peak group whose intensity was more than 1 000 000, *m*/*z* values for the highest intensity signals were not used for the mean value calculation. Instead, a mean value was calculated using the *m*/*z* values of mass peaks whose intensities were just below 1 000 000. Molecular formulae that matched a given accurate *m*/*z* value were determined as follows. A library of molecular formulae with all possible elemental combinations whose theoretical *m*/*z* matched the input *m*/*z* with 1 ppm tolerance was generated using elements C, H, N, O, P and S. To screen the library for chemically possible molecular formulae, all formulae were tested for whether they met following criteria (Senior, 1951): (i) the sum of valences is an even number, and (ii) the sum of valences is greater than or equal to twice the number of atoms minus 1. The accurate *m*/*z* was used for molecular formula calculation. Upper limits of 95 for C, 182 for H, 10 for N, 45 for O, 6 for P and 5 for S were used for calculation of formulae. In addition, the relative intensity of the ^13^C_1_ isotopic ion was calculated. The number of carbons in the molecular formula was estimated using the following equation: 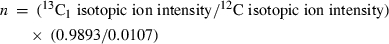
where *n* represents the number of carbons. The tolerance for relative intensity was set at 5%. Chemically possible molecular formulae and the relative intensities of the isotope ions were calculated by programs written in Java. The library of molecular formulae was constructed using MySQL. A Java program was developed to search the molecular formula library for molecular formulae matching the criteria described above. Any peak group that is selected based on these criteria is defined as a metabolite. The analysis was repeated three times for each tomato fruit tissue. When a metabolite was detected in two or more repeats, it was regarded as ‘present’ in that tissue. Computational assignment of peak groups of isotopic ions to the parental metabolite was re-checked manually. Assignments of fragment ions and adduct ions to the parental metabolite were performed manually. Peak groups composed of adduct ions produced during ionization were assigned using two criteria as follows. First, it was checked whether the *m*/*z* values of ions matched theoretical *m*/*z* values of adducts ([M+Na]^+^, [M+K]^+^, [M+NH_3_+H]^+^, [M+CH_3_CN+H]^+^ (Svatos *et al.*, 2004) and [2M+H]^+^). Second, retention time was checked to determine whether the adduct ions co-eluted with the proton adduct ion. In negative-ionization ESI mode, formic acid adduct ions ([M+HCOO]^−^) were frequently produced together with [M-H]^−^ ions, and were assigned using the same criteria. Metabolite annotations were provided for the adduct ion species with the highest intensity, i.e. [M+H]^+^ and [M-H]^−^ in positive- and negative-ionization ESI modes, respectively, for the majority of the metabolites detected in the present study (Table S2). After these manual curation processes, metabolites were designated as ‘annotated metabolites’.

### Database construction

For database construction, a dataset comprised of accurate *m*/*z* values, predicted molecular formula, retention time, MS/MS data and λ_max_ of the UV/visible absorption spectra was compiled. As MS/MS data, the *m*/*z* value, raw intensity and relative intensity of the 20 highest-intensity MS/MS fragment ions were retrieved. References for each annotated metabolite were searched for in the public databases PubChem (http://pubchem.ncbi.nlm.nih.gov/), the Dictionary of Natural Product (http://www.chemnetbase.com/scripts/dnpweb.exe?welcome-main), KNApSAcK (http://kanaya.naist.jp/KNApSAcK/), KEGG (http://www.genome.jp/kegg/kegg2.html) and MotoDB (http://appliedbioinformatics.wur.nl/moto/). To browse and search the annotation information, a web-based database (http://webs2.kazusa.or.jp/komics/) was constructed using MySQL and PHP.

### Annotation grading system

To each metabolite, an annotation grade was added to describe the evidence supporting the annotations for that metabolite (Figure 2). First, annotations were classified into two grades (A/B versus C) according to whether a single molecular formula was obtained or not. Grades A and B were further classified according to whether the mass spectral attributes of the metabolites matched those of standard chemicals or not. In grade A, annotations were verified by comparison with standard chemicals. In grade B, annotations were assigned with single molecular formulae but lacked verification by standard chemicals. Annotations in grade B were classified into eight sub-grades according to the availability of MS/MS, λ_max_ and reference information. In grade C, multiple molecular formulae were assigned to each metabolite. Annotations in grade C were classified into eight sub-grades according to the availability of MS/MS and λ_max_ information.

### Mass difference analysis

Mass difference values (Δ[*m*/*z* ]) were calculated for pairwise combinations of *m*/*z* values shown in Table S2 at the 0.001 Da interval. Δ[*m*/*z* ] values were calculated separately for *m*/*z* datasets of tomato tissue samples and for *m*/*z* datasets obtained in positive- and negative-ionization ESI modes. Δ[*m*/*z* ] values were calculated in the 500 Da range. To identify Δ[*m*/*z* ] values that occurred more frequently than others, a threshold probability was determined based on the standard deviation of the probability distribution within each sample. Probabilities of 10-, 20-, 30-, 40-, 50-, 60- and 70-fold standard deviation levels were tested, and the 40-fold standard deviation level was used as the threshold (Figure S1). MS/MS data inspection was performed manually using *m*/*z* values for the 20 fragment ions with highest intensity. To match MS/MS fragments between a pair of metabolites, the *m*/*z* tolerance was set to 0.1% as MS/MS spectra were obtained by the ion-trap mode, which is less accurate than the FTICR mode. A pairwise difference in elemental composition was calculated based on the molecular formula annotation provided in Table S2.Δ[*m*/*z* ] spikes between stages were identified using following criteria: (i) the probability was above the 40-fold standard deviation level, (ii) the frequency of the Δ[*m*/*z* ] value increased in the later stages, and (iii) the probability of the Δ[*m*/*z* ] value increased in the later stages. To obtain the chemical information of KEGG compounds, compound files were first retrieved from the KEGG ftp site (ftp://ftp.genome.jp/pub/kegg/ligand/compound/, 9 March 2007), and then compounds containing C, H and O in the molecular formula were selected. Finally, compounds with a non-redundant compound ID were chosen. The theoretical molecular weight of KEGG compounds were calculated using accurate masses of the elements C, H, N, O, P and S. Programs for calculating Δ[*m*/*z* ] and elemental composition difference were written in Perl. The program for the selection of KEGG compounds was written in Java.

## Supplementary material

Figure S1 (A) The numbers of Δ[m/z] spikes detected at different threshold probability. Δ[m/z] spikes were detected in each of eight tomato fruit samples. The plotted points indicate means and SE of eight samples. (B) Decrease ratio of the number of Δ[m/z] spikes. “Decrease level” is defined as follows: [Decrease level at (n×10)-fold standard deviation threshold] = [The number of spikes detected at (n×10)-fold standard deviation threshold] / [The number of spikes detected at {(n-1)×10}-fold standard deviation threshold]. The arrow indicates an inflection point at the 40-fold standard deviation level. (C) Coefficient of variance of the numbers of Δ[m/z] spikes at different threshold probability. The arrow indicates the maximal point at the 40-fold standard deviation level. The number of spikes identified in each sample decreased with increase of threshold level (Panel A). A decrease-trend curve had an inflection point at 40-fold standard deviation level (Panel B). Additionally, coefficient of variance for the number of spikes showed maximal value at 40-fold standard deviation level, implying that considerable sample-to-sample variation of spike numbers was expected at this threshold (Panel C). Thus, we used a threshold of 40-fold standard deviation values to identify Δ[m/z] spikes.

Figure S2 Tissue-dependent occurrence of Δ[m/z] spikes. The numbers in boxes indicate Δ[m/z] values. (A) Δ[m/z] spikes detected in both peel and flesh samples. (B) Δ[m/z] spikes specifically detected in peel samples. (C) Δ[m/z] spikes detected preferentially in flesh samples. Abbreviations for tissues: GP, mature green peel; BP, breaker peel; TP, turning peel; RP, red peel; GF, mature green flesh; BF, breaker flesh; TF, turning flesh; RF, red flesh. P(Δ[m/z]) indicates probability of the occurrence of Δ[m/z] values.

Table S1 *m*/*z* values for five major metabolites in tomato fruits before and after the internal standard calibration.

Table S2 Mass spectral data and metabolite annotations of the metabolites in Micro-Tom fruit tissues.

Table S3 Molecular formulae and molecular weights of KEGG CHO compounds.

Table S4 Δ[*m*/*z*] spikes at the threshold probability of 40-fold standard deviation level.

Table S5 Flavonoids in Micro-Tom fruit tissues.

Table S6 Glycoalkaloids in Micro-Tom fruit tissues.
