# Supplementary material for: Metabolite annotations based on the integration of mass spectral information
Source: Plant J. 2008 Apr 14;54(5):949–62. doi: 10.1111/j.1365-313X.2008.03434.x (PMC2440531; doi:10.1111/j.1365-313X.2008.03434.x)
Supplement: Table S1 — m/z values for five major metabolites in tomato fruits before and after the internal standard calibration. [file tpj0054-0949-SD3.doc]

**Supplementary Table S1.** m/z values of five major metabolites in tomato fruits before and after the internal standard calibration.

| Metabolite | Molecular formula | Theoretical [M+H]^+^ | Detected [M+H]^+a^ | | I.S. recalibrated [M+H]^+ b^ | |
| --- | --- | --- | --- | --- | --- | --- |
|  |  | m/z | m/z | Error  (ppm) | m/z | Error (ppm) |
| Citric acid | C_6_H_8_O_7_ | 193.03428 | 193.03433 | 0.26 | 193.03423 | -0.26 |
| Naringenin chalcone | C_15_H_12_O_5_ | 273.07574 | 273.07588 | 0.51 | 273.07588 | 0.51 |
| Sucrose | C_12_H_22_O_11_ | 343.12349 | 343.12362 | 0.38 | 343.12358 | 0.26 |
| Rutin | C_27_H_30_O_16_ | 611.16066 | 611.16145 | 1.29 | 611.16081 | 0.25 |
| Tomatine | C_50_H_83_NO_21_ | 1034.55303 | 1034.55449 | 1.41 | 1034.55247 | -0.54 |

^a^m/z values before internal standard (I.S.) calibration. ^b^m/z before internal standard (I.S.) calibration.
